# Sustainability of the regional financial system: a case study of the Northwestern Federal District

**DOI:** 10.12688/f1000research.123197.1

**Published:** 2022-08-08

**Authors:** Sergey Evgenievich Barykin, Alexey Aleksandrovich Mikheev, Elena Grigorievna Kiseleva, Yuriy Evgenievich Putikhin, Elena De La Poza Plaza, Natalia Sergeevna Alekseeva

**Affiliations:** 1 Graduate School of Service and Trade, Peter the Great St. Petersburg Polytechnic University, St. Petersburg, Russian Federation; 2 Moscow State Institute of International Relations (University), Ministry of Foreign Affairs of the Russian Federation, Moscow, Russian Federation; 3 Graduate School of Industrial Management, Peter the Great St. Petersburg Polytechnic University, St. Petersburg, Russian Federation; 4 Department of Management, Financial University under the Government of the Russian Federation, Moscow, Russian Federation; 5 Center for Economic Engineering (INECO), Universitat Politècnica de València, Valencia, Spain

**Keywords:** Financial Stability; Integral Index; Regional Financial System; Regions of Russia; Indicators; The Northwestern Federal District

## Abstract

**Background:** This article provides an assessment of the sustainability of Russian regions’ financial systems. The study is based on the methods of generalization and synthesis, correlation-regression analysis, and multivariate classification. Since the structure of the regional financial system is complex, several works are devoted to studying its sustainability issues. The relevance of the study topic is confirmed by the lack of a systematic approach to assessing the integral index of sustainability and the possibility of using various tools in determining the complex indicator.

**Methods:** This  methodology with application of mathematical statistics methods makes it possible to assess the financial system sustainability in four sectors, to include the leading indicators in the assessment, and to identify regions with extreme values of debt burden indicators. The method was tested for the regions of the Northwestern Federal District (NWFD) for the period 2010 - 2019  to classify the regions according to three levels of debt sustainability. Data collection from the 1 ^st^ January to 30 ^th^ April 2022 included statistical data from government open internet sources, sectors studied relate to government, and municipal budgets in the NWFD. Authors analyzed regional debt sustainability indicators and identified themes in the field of sustainability studies for the NWFD.

**Results:** An increased level of financial system sustainability was observed among the  NWFD regions in the corporative and personal finance sectors, indicating a significant contribution of businesses and households to maintaining the balance and sustainability of the financial system in Russia as a whole. The results of the study also identified that the NWFD regions belong to three clusters: cluster 1 - high debt sustainability; cluster 2 - medium debt sustainability; and cluster 3 - low debt sustainability.

**Conclusions:** The study results allowed the identification of regions with a constantly high level of debt, financial, and corporative sustainability.

## Introduction

Financial stability is one of the critical tasks in the functioning of the financial system of a country. National financial systems have significant differences in their development, structure, and approaches to regulation. The issues of assessing sustainability and stability of financial systems are relevant for different countries ( Mazanec and Bartosova, 2021; Străchinaru and Dumitrescu, 2019; Jiang *et al*., 2019; Santos, Lisboa, and Eugénio, 2021; Chupina *et al*., 2021). The use of financial resources in various sectors of the economy, on the one hand, contributes to an increase in investment activity and an increase in the stability of the financial system, but, on the other hand, it may lead to its destabilization in case of low and untimely financing of its various sectors. In this regard, the issues of assessing the sustainability of regional financial systems are the subject of debate ( Schinasi, 2004) and are actively discussed by the world scientific community ( Schellhorn, 2020; Checherita-Westphal, Hughes Hallett, and Rother, 2014; Baharumshah, Soon, and Lau, 2017; Ghosh and Sahu, 2021).

During the study, the authors concluded that changes in financial stability could not be summarized in one quantitative indicator. Unlike, for example, price stability, there is still no unambiguous unit for measuring financial stability. This reflects the multifaceted nature of the financial system, related to financial institutions' resilience and sustainability and the smooth functioning of financial markets and settlement systems. Therefore, the authors divided financial system stability into four sectors: the public finance sector, which characterizes the development of the budget system and its debt sustainability; the financial sector, which represents the state and sustainability of the banking sector, the insurance sector, the non-state pension funds, microfinancing entities, and cooperatives; the corporative sector, which takes into account the stability of cash flows of small businesses and the scale of large regional enterprises; and the personal finance sector, which takes into account the contribution of the households to the stable functioning of the financial system.

We would like to note that changes in financial stability are inherently difficult to predict. The state of financial system sustainability should assess violations as they occur and indicate the risks and vulnerabilities that may lead to such violations in the future. Therefore, an integrated approach is necessary to determine the growth of risks and imbalances and consider the time lag when using the integral financial system sustainability index to develop the public financial policy.

Besides the time delays, the policy instruments of financial stability are often indirect. In some cases, disagreements may occur on the original purpose of the device and the implications of its application for the stability of the regional financial system. At the same time, policies aimed at financial stability often involve a trade-off between sustainability and efficiency. Measures to improve financial stability include, on the one hand, striving for efficient allocation of financial resources, and on the other hand, are designed to reduce the risks or vulnerabilities of the financial system. So, for example, foreign exchange restrictions can reduce or eliminate certain risks associated with international capital flows while reducing the efficiency of the domestic financial market ( He, Korhonen, and Qian, 2021; Gurrea-Martínez, 2021; Rendon, 2019).

### Literature review

The issues of assessing financial stability and vulnerability of financial systems are widely discussed in the scientific literature. For example, in Khalatur *et al.*’s study (2021), the method of deriving regression equations in multivariate regression analysis is used to test the model for assessing the financial system's sustainability. Based on the data from several Eastern European countries from 2010 to 2019, a model of the modern business space ‘Volatility, uncertainty, complexity and ambiguity’ (VUCA) was developed on the example of the banking sector. The driving forces, consequences, requirements, and macroeconomic indicators of countries’ activities under the conditions of ‘VUCA-world’ were determined. The analysis showed that with an increase in GDP growth factors, growth in gross national income per capita, research and development costs, foreign direct investment, and net capital inflows by 1%, the effective ratio of the bank capital to the assets also increases by 1%.

The scientific works also note that there are no uniform worldwide standards, methods, or indicators for assessing financial sustainability ( Babar *et al*., 2019; Nasreen and Anwar, 2019; Jadoon *et al.,* 2021; Shkolnyk *et al.,* 2021). A broad scope is employed in order to evaluate and test system sustainability based on different indicators and standards. The results obtained in the scientific works are diverse and interesting in terms of data coverage and other assessment methods. So, for example, in the study by Ukrainian scientists ( Shkolnyk *et al.,* 2021) on big data related to the structure of the financial system of Ukraine, a matrix of the financial system sustainability characteristics was developed, according to the 4x2 principle proposed by experts of the International Monetary Fund ( Schinasi, 2004). The list of indicators for calculating the integral indicator characterizing the stability of the financial system of Ukraine covers the period from 2007 to 2019. It includes 29 indicators that consider the peculiarities of its formation and development. Harrington’s desirability function is used to determine an integral indicator that characterizes the state of financial stability. As a result, intermediate calculations obtained by modeling groups of indicators showed that the level of access to the financial system and its depth were balanced during the study period. The financial system’s efficiency is low and characterized by high volatility (the range of variations is 0.51).

An important aspect is a statement made by the scientists that it is essential to study the stability of the financial system from the point of view of resistance to a crisis ( Gustiana and Nasrudin, 2021; Albulescu, 2010). The paper substantiates the thesis that instability faced by the countries is a consequence of the vulnerability of the financial system. As a method for assessing the sustainability of the financial system, the aggregate financial index (AFSI) is used, which takes into account the influence of other countries on the stability of the Indonesian banking system, and the Indonesian financial system stability index (ISSK), which takes into account the internal stability of the banking system of this country. Using the principal component analysis (PCA) method, one of the multivariate statistical analysis methods, the authors established specific weights for each index, which allowed to refine the index values for the period from 2000 to 2019. As a result, the paper concludes that each period of a crisis is always accompanied by pressure from the global economic environment, which significantly affects the sustainability of the regional financial system in Indonesia. The index developed by the scientists will serve as the basis for changing the regional policy of the Indonesian authorities.

Also, the study separately raises questions on the quality of the banks’ accounting, which plays a decisive role in assessing the financial sustainability of the banking system ( Acharya and Ryan, 2016; Arzamasov and Penikas, 2014). The world scientific community is coming to understand the high role of transparency and openness of accounting information as a guarantee of objectivity in assessing the financial system sustainability. The scientists actively discuss how banking accounting and information disclosure can affect the assessment of the financial system's sustainability. The articles pay particular attention to recommendations for developers of accounting standards and policymakers of the financial system ( Badertscher, Burks, and Easton, 2012; Grassa, Moumen, and Hussainey, 2021; Acharya and Ryan, 2016).

## Methods

### Methodology for assessing the debt sustainability of the regional budget system

**Figure 1.**
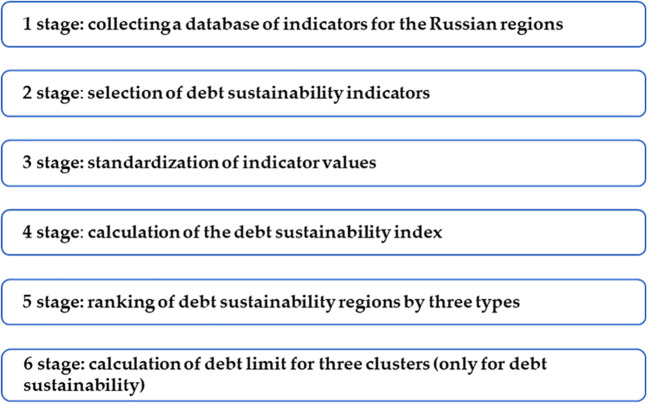
Methodology for assessing the sustainability of the financial system.

The assessment of the sustainability of the financial system of the regions of the Russian Federation is carried out using the author’s methodology, which is a set of stages and assessment methods and a specific algorithm for their application. The developed method assumes: a) justification of choice and systematization of indicators of the debt sustainability, financial sustainability, corporative sustainability, and personal sustainability; b) standardization of the indicator values based on the method of Euclidean distances; c) calculation of the integral indicator in each sustainability sector, its ranking using the multivariate mean formula; d) division of regions into three sustainability groups: low, medium and high; and e) determination of the limit values (threshold values, limits) for the debt sustainability group using the hierarchical cluster analysis ( Figure 1).

At the first stage of the study, using open data from the Federal State Statistics Service, a database of the Russian economy's statistical financial and economic indicators was formed. Next, a calculation of about 45 indicators of the financial system sustainability in four sectors was carried out. Then, at the second stage of the study, using the correlation analysis method, six indicators of debt sustainability, nine financial sustainability indicators, seven corporative sustainability indicators, and four personal sustainability indicators were selected. All indicators were highly correlated with the integral sustainability index for each financial system sector, respectively.

At the third stage of the study, all indicator values were standardized. To consider the degree of differences of each indicator across the federal districts, the method of Euclidean distances was applied. At the fourth stage of the study, the standardized values of six indicators were averaged over a dynamic series using the multivariate mean formula. A composite index (an integral indicator) across four sectors was calculated for each region of the Northwestern Federal District (NWFD). At the fifth stage of the study, a hierarchical cluster analysis was carried out, and the limit boundaries were obtained for the six indicators for the debt sustainability sector. Control of budget constraints within the boundaries found by us will increase the debt sustainability of the budget system of the Russian regions. We also used a distance matrix of indicators over the regions of the NWFD for each analyzed period and divided the data set into three clusters according to the type of debt, financial, corporative, and personal sustainability: low, medium, and high.

### Study design

The study is based on the open data from the Federal State Statistics Service and the Ministry of Finance of the Russian Federation: 53 absolute indicators (the most typical indicators for the mentioned regions) of the socio-economic development of Russian regions were selected for the period from 2010 to 2019 (in 11 regions of the Northwestern Federal District (NWFD) which includes: Republic of Karelia, Republic of Komi, Arkhangelsk region, Vologda region, Kaliningrad region, Leningrad region, Murmansk region, Novgorod region, Pskov region, Saint Petersburg, Nenets Autonomous region), such as the local government debt, the volume of exports and imports, the gross domestic product, the population, the income of people per capita, the expenses for repayment and servicing of the local government debt, the amount of insurance premiums and payments, the level of the overdue debt on loans, the amount of deposits, the amount of loans granted to individuals and legal entities, the actual average wages, the consumer price index, the amount of payable and receivable accounts of enterprises, the profit of enterprises, etc. On the basis of the absolute indicators, 26 relative indicators of sustainability were calculated (for the calculations please see Barykin *et al.,* 2022) across four sectors (the public finance sector, the financial sector, the corporative sector, and the personal finance sector) for 11 regions of the NWFD. Authors calculated indices for the debt, financial, corporative, and personal sustainability. The study uses the methods of comparison and grouping, correlation analysis, Euclidean distances, calculation of the multivariate mean, and hierarchical cluster analysis. Selection of the sustainability indicators was carried out using the correlation analysis, which has proven itself as a method for studying the relationship between the debt sustainability of the budget system and the risk of the government debt ( Eberhardt and Presbitero, 2015; Égert, 2015; Li *et al.,* 2021). Data on the selected indicators were collected from 1 ^st^ January 2022 to 30 ^th^ April 2022. The open service of the Federal State Statistics Service contains both separate information on statistical indicators and statistical reports for different time periods. Statistical reports used for this study were ‘Russia in numbers’ ( https://fedstats.ru/, accessed 30 ^th^ March 2022) and’Regions of Russia’ ( Federal State Statistics Services, n.d.-b) to collect financial data while socio-economic indicators were collected from the’Russian Statistical Yearbook’ ( Federal State Statistics Services, n.d.-c). Translation of all reports from Russian to English were undertaken by the authors in this study. In order to get data from 2010 to 2019, these reports were collected for various years. In the process of collecting information, some variables were excluded because they were partially presented, since it was not possible to find data for each year from the study period (the data from the period from the year of 2010 till the end of the year of 2015 has been excluded for the purpose of making conclusions regarding regional comparisons). Figure 1 shows the stages methodology for assessing the sustainability of the financial system.

### Data analysis

The calculation of the integral sustainability indicator was carried out using the multivariate mean formula, which has proven itself for ranking indicators of digital potential ( Kiseleva, 2020). To determine the budget constraints for the debt sustainability sector for each region of the NWFD, the hierarchical cluster analysis was applied since it is one of the methods of multivariate classification that allows to select areas of congestion of objects from the aggregate data and to combine them into groups (segments) with homogeneous characteristics ( Kurushin and Vasilyeva, 2017). The authors used the method of Euclidean distances and cluster analysis to standardize the obtained values. Both methods are widely used in natural and social sciences to standardize and classify panel data ( Barykin *et al*., 2021b; Bagirova and Shubat, 2021; Zolkover *et al*., 2020). In studying debt sustainability, using a distance matrix of indicators over the regions of the NWFD for each analyzed period, we divided the data set into three clusters: cluster 1 - high debt sustainability; cluster 2 - medium debt sustainability; and cluster 3 - low debt sustainability. Then the authors identified the cluster centroids for each indicator. Cluster centroids were determined based on the values of cluster centroids of indicators for each year of the study period, taking into account their average number, as well as the dynamics of change over the last four periods (the year of 2016, the year of 2017, the year of 2018, and the year of 2019). This allowed confirming the results obtained at the previous stage of the study and solve the problem of targeted statistical justification of the norms of budget restrictions on the debt burden. On the one hand, the values of cluster centroids were compared with the actual values of debt sustainability indicators, which allowed us to confirm the results obtained earlier. On the other hand, using clustering, the criteria values of debt sustainability indicators were justified. As a method of clustering, we used the method of intergroup communication, and as a measure of the similarity between objects, we used the Euclidean distance. The application of the author’s methodology using the standardization of indicator values and the multivariate mean formula allowed the authors of the study to rank the regions according to the level of the debt, financial, corporative, and personal sustainability, and the use of the hierarchical cluster analysis allowed to group the regions by three types of the debt sustainability and to determine the acceptable boundaries of the debt sustainability indicators for each cluster. All calculations in our study were performed using MS Excel 2016 for calculations.

## Results

### Evidence of Russian regions

The debt sustainability indicators included were: the ratio of the government debt to gross regional product (GRP); the amount of the local government debt on per capita; the share of the government debt in regional export; the ratio of the government debt to total budget revenues; the percentage of expenditures on servicing of the government and municipal debt in regional budget expenditures; the balance of annual payments for servicing and repayment of the government debt to total budget revenues. The last three indicators refer to the regulated indicators of the Budget Code of the Russian Federation ( Federal State Statistics Services, n.d.-a).

The nine financial sustainability indicators included were: the ratio of deposits of legal entities to the number of registered enterprises and organizations; the deposits of individuals per capita; the ratio of loans granted to legal entities to the number of registered enterprises and organizations; the loans given to individuals per capita; the amount of debt on loans of legal entities and individuals to the loans granted; the volume of overdue debt on loans of legal entities to the number of registered enterprises and organizations; insurance premiums; and insurance payments.

The sector of corporative finance (business statistics) is represented by seven indicators: profitability of enterprises; unprofitability of enterprises; accounts receivable turnover; accounts payable turnover; the ratio of overdue accounts payable to revenue; the ratio of overdue receivables to revenue; and the share of unprofitable organizations in the total number of organizations.

The sector of personal finance (population statistics) includes four indicators: average per capita money income; average monthly wages; the average amount of assigned pensions; average per capita consumer expenditures of the population; and overdue wage arrearages.

Thus, the values of the indicators were calculated for the regions of the NWFD for the period from 2010 to 2019 ( Barykin *et al.,* 2022). A fragment of the obtained results for 2016-2019, containing sample data by years for the debt sustainability sector, is provided in Table 1. Full results are available in the underlying data in the index ids tab ( Barykin *et al.,* 2022). Similar tables were calculated for the other sectors but are not provided here because of the limited scope of the work ( Barykin *et al.,* 2022). According to Table 1, we can see that two regions stand out from the others by the low values of the indicators, allowing us to preliminarily classify them as regions with a high level of debt sustainability: Saint Petersburg and the Leningrad Region (LR). High values of the indicators for the analyzed period are most often observed in the Republic of Karelia (RKA), the Republic of Komi (RKO), the Vologda (VR), Arkhangelsk (AR), and Pskov regions (PR), therefore, most likely, these regions of the Russian Federation will be classified as regions with a low level of debt sustainability.

**Table 1.** Partial results of the study: debt sustainability indicators of Northwestern Federal District’s (NWFD) financial system (by the authors’ calculations).

Period	NWFD * regions											
	NWFD *	RKA *	RKO *	AR *	VR *	KR *	LR *	MR *	NR *	PR *	SPb *	NAR *
**Indicator 1:** ratio of government debt to GRP ^+^, %												
2016	3.08	**10.04**	6.39	9.36	7.17	5.82	1.12	5.15	6.6	9.84	**0.44**	**0.44**
2017	3.01	9.92	6.7	8.76	4.68	5.41	**0.41**	4.29	6.24	**10.81**	0.91	1.28
2018	2.25	6.15	4.3	5.71	3.89	3.5	0.33	3.86	5.98	**9.48**	0.79	**0.66**
2019	2.1	5.82	3.53	**6.08**	2.49	4.19	**0.23**	2.28	5.68	8.64	0.59	0.48
**Indicator 2:** amount of government debt on per capita, rub/person												
2016	16.7	36.1	49.2	36.6	26.2	21.8	34.6	27.1	24.9	22.9	**2.63**	**81.8**
2017	17.50	39.99	45.58	36.65	20.19	22.80	**2.17**	25.12	25.85	25.61	6.55	**75.63**
2018	14.5	27.8	34.2	26.5	19.3	16.2	**1.9**	24.8	26	24.6	6.2	**45.54**
2019	14.1	30.7	30.9	31	13.5	21.6	**1.5**	18.9	25.9	27.1	5.6	**36.4**
**Indicator 3:** share of government debt in regional export, %												
2016	12.03	51.9	87.97	30.44	18.58	27	2.02	12.68	27.43	**409.12**	**1.48**	0
2017	11.35	41.74	79	30.59	14.74	32.26	**1.28**	9.61	28.01	**491.86**	3.13	0
2018	9.01	160.13	241.63	218.23	64.85	3.21	**1.46**	90.59	61.74	94.46	2.43	0
2019	6.81	31.08	46.3	22.15	6.76	26.08	**0.71**	5.41	16.7	**277.42**	1.93	4.01
**Indicator 4:** ratio of government debt to total budget revenues excluding UCR, %												
2016	23.92	**79.35**	63.14	67.4	54.44	50.91	4.83	31.2	55.34	74.91	**2.98**	27.53
2017	23.55	**90.41**	50.44	61.88	37.94	50.98	**3.11**	28.89	59.12	78.3	6.95	17.98
2018	14.72	37.32	37.35	35.99	**171.68**	13.98	**2.27**	25.58	66.04	131.87	5.63	8.6
2019	15.21	53.91	28.17	41.65	18.67	39.02	**1.71**	15.88	51.01	**71.58**	4.85	7.63
**Indicator 5:** share of expenditures on servicing of government and municipal debt in regional budget expenditures, %												
2016	1	2.9	2.6	1.37	1.63	0.38	1.42	1.34	3.3	**3.02**	**0.1**	1.8
2017	1.06	2.52	6.1	1.89	1.06	0.29	1.52	0.8	1.57	**2.8**	**0.04**	1.55
2018	1.22	1.77	3.75	1.41	0.61	0.23	**3.51**	0.77	1.08	2.23	**0.4**	2.83
2019	1.37	1	4.61	0.67	**0.16**	0.21	**5.5**	0.9	0.97	2.22	0.35	2.57
**Indicator 6:** ratio of annual payments for servicing and repayment of government debt to total budget revenues excluding UCR, %												
2016	14.6	25.6	55.47	51.8	21.03	28.57	1.53	26.09	22.3	**59.25**	**0.32**	28.16
2017	17.66	50.54	55.87	69.01	17.36	23.49	2.72	34.91	20.57	**69.88**	**1.85**	20.16
2018	20.35	47.46	44.87	102.51	134.46	22.98	3.52	65.38	42.88	**162.67**	**1.2**	26.84
2019	19.36	64.77	14.71	**108.78**	15.79	24.19	6.02	68.93	16.69	80.67	**0.37**	7.12

* Abbreviations: NWFD - Northwestern Federal District, RKA - Republic of Karelia, RKO - Republic of Komi, AR - Arkhangelsk region, VR - Vologda region, KR - Kaliningrad region, LR - Leningrad region, MR - Murmansk region, NR - Novgorod region, PR - Pskov region, SPb - Saint Petersburg, NAR - Nenets Autonomous region, UCR - uncompensated receipts.

+ GRP - gross regional product.

** The maximum and minimum values of the indicators are highlighted in bold.

Standardization of the indicator values using the method of Euclidean distances and further ranking of the resulting base of standardized estimates using the multivariate mean formula made it possible to obtain the debt, financial, corporative, and personal sustainability indices for each region of the NWFD ( Tables 2– 5). The use of the multivariate mean formula in our study correlates the value of a standardized indicator with the average value for the regions in the assessed year and allows us to identify the regions where the level of sustainability is significantly higher than the average value, close to the average value and significantly lower than the average value.

**Table 2.** Results of the debt sustainability study for the Northwestern Federal District (NWFD) (by the authors’ calculations).

NWFD regions	Standardized indicator values												Debt sustainability index, I _DS_			
	2019						2018									
	I1	I2	I3	I4	I5	I6	I1	I2	I3	I4	I5	I6	2019	2018	2017	2016
Republic of Karelia	0.04	0.05	0.02	0.06	0.16	0.01	0.05	0.07	0.01	0.06	0.13	0.03	0.24	0.27	0.22	0.19
Republic of Komi	0.07	0.24	0.02	0.04	0.03	0.03	0.08	0.06	0.01	0.06	0.06	0.03	0.32	0.23	0.19	0.18
Arkhangelsk region	0.04	0.12	0.03	0.09	0.24	0.01	0.06	0.07	0.01	0.06	0.16	0.01	0.36	0.28	0.26	0.25
Vologda region	0.09	0.09	0.11	0.04	1	0.02	0.08	0.10	0.02	0.01	0.38	0.01	0.88	0.42	0.45	0.32
Kaliningrad region	0.05	0.12	0.03	1	0.76	0.02	0.09	0.12	0.45	0.16	1.00	0.05	**1.55**	**1.36**	**1.32**	0.53
Leningrad region	1	0.44	1	0.11	0.03	0.06	1.00	1.00	1.00	1.00	0.07	0.34	**2.20**	**3.78**	**3.45**	**2.53**
Murmansk region	0.1	0.16	0.13	0.03	0.18	0.01	0.09	0.08	0.02	0.09	0.30	0.02	0.44	0.42	0.45	0.41
Novgorod region	0.04	0.13	0.04	0.02	0.16	0.02	0.06	0.08	0.02	0.03	0.21	0.03	0.29	0.32	0.32	0.27
Pskov region	0.03	0.69	0.01	0.35	0.07	0.005	0.03	0.08	0.02	0.02	0.10	0.01	0.82	0.20	0.55	0.19
Saint Petersburg	0.39	1	0.37	0.22	0.46	1	0.42	0.32	0.60	0.40	0.58	1.00	**3.22**	**2.97**	**3.41**	**5.70**
Nenets AR	0.48	0.04	0.18	0.01	0.06	0.05	0.50	0.04	0.00	0.26	0.08	0.04	0.68	0.74	0.37	0.41
Mean value	0.21	0.28	0.18	0.18	0.29	0.11	0.22	0.19	0.2	0.2	0.28	0.14	1.00	1.00	1.00	1.00

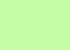
 - Three maximum values of indicators are highlighted.

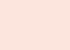
 - Three minimum values of indicators are highlighted.

Abbreviation: NWFD - Northwestern Federal District.

**Table 3.** Results of the financial sustainability study for the Northwestern Federal District (NWFD) (by the authors’ calculations).

NWFD regions	Standardized indicator values									Financial sustainability index, I _FS_			
	2019												
	I1	I2	I3	I4	I5	I6	I7	I8	I9	2019	2018	2017	2016
Republic of Karelia	0.012	0.219	0.351	0.68	0.905	0.84	0.295	0.465	0.445	0.81	0.86	0.78	0.65
Republic of Komi	0.008	0.391	0.7	0.632	1	0.921	0.268	0.569	0.518	0.99	0.96	1.09	1.19
Arkhangelsk region	0.015	0.383	0.302	0.759	0.502	0.934	1	1 *	0.885	1.23	1.19	1.04	0.99
Vologda region	0.895	0.341	0.223	0.577	0.713	0.941	0.249	0.974	0.999	1.46	1.84	1.61	1.4
Kaliningrad region	0.021	0.475	0.35	0.671	0.407	1	0.146	0.901	1	0.93	0.85	0.88	0.98
Leningrad region	0.019	0.216	0.524	0.746	0.509	0.763	0.059	0.929	0.815	0.84	0.86	0.93	0.93
Murmansk region	0.086	0.545	0.402	0.871	0.363	0.901	0.158	0.655	0.847	0.95	0.86	0.88	0.83
Novgorod region	0.008	0.27	0.4	0.534	0.66	0.925	0.113	0.406	0.372	0.69	0.57	0.6	0.69
Pskov region	0.012	0.251	0.178	0.041	0.448	0.97	0.433	0.241	0.224	0.58	0.51	0.5	0.49
Saint Petersburg	1	1	1	1	0.64	1.007	0.11	1	1	1.92	1.58	1.8	2.06
Nenets AR	0	0.485	0.122	0.772	0.423	0.772	0.368	0.012	0.018	0.61	0.92	0.89	0.79
Mean value	0.19	0.42	0.41	0.66	0.6	0.91	0.29	0.65	0.65	1	1	1	1

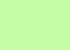
 - Three maximum values of indicators are highlighted.

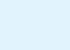
 - The minimum values of indicators are highlighted.

* Since when calculating the indicators I8, I9 a high differentiation of the NWFD regions is observed in relation to the city of Saint Petersburg, the latter was forcedly assigned a high sustainability level (1), and further ranking was carried out without taking Saint Petersburg into account.

Abbreviation: NWFD - Northwestern Federal District.

**Table 4.** Results of the corporative sustainability study for the Northwestern Federal District (NWFD) (by the authors’ calculations).

NWFD regions	Standardized indicator values							Corporative sustainability index, I _СS_			
	2019										
	I1	I2	I3	I4	I5	I6	I7	2019	2018	2017	2016
Republic of Karelia	0.597	0.090	0.759	0.367	0.332	0.023	0.140	0.84	0.7	0.8	0.7
Republic of Komi	0.192	1.000	0.536	0.994	0.365	0.220	0.156	1.13	1.0	1.0	1.0
Arkhangelsk region	0.233	0.331	0.171	0.068	0.046	0.128	0.168	0.43	0.4	0.5	0.5
Vologda region	0.284	2.616	1.000	1.000	0.433	0.408	0.201	1.85	2.0	1.5	1.6
Kaliningrad region	0.107	0.479	0.590	0.515	0.999	1.000	1.000	1.99	1.9	2.0	1.6
Leningrad region	1.000	0.023	0.098	0.104	0.163	0.293	0.196	0.84	0.9	0.9	0.9
Murmansk region	0.376	0.088	0.378	0.288	0.154	0.049	0.160	0.56	0.8	0.8	0.9
Novgorod region	0.202	0.498	0.654	0.880	0.286	0.390	0.138	1.04	1.1	0.9	1.0
Pskov region	0.116	0.673	0.694	0.620	0.448	0.302	0.172	1.03	1.0	1.0	1.1
Saint Petersburg	0.122	0.366	0.366	0.378	0.275	0.316	0.275	0.79	0.8	0.8	0.9
Nenets AR	0.246	0.086	0.688	0.237	0.000	0.100	0.120	0.50	0.4	0.8	0.8
Mean value	0.32	0.57	0.54	0.50	0.32	0.29	0.25	1.00	1.00	1.00	1

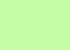
 - Three maximum values of indicators are highlighted.

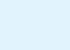
 - The minimum values of indicators are highlighted.

Abbreviation: NWFD - Northwestern Federal District.

**Table 5.** Results of the personal (household statistics) sustainability study for the Northwestern Federal District (NWFD) (by the authors’ calculations).

NWFD regions	Standardized indicator values								Personal sustainability index, I _РS_			
	2019				2018							
	I1	I2	I3	I4	I1	I2	I3	I4	2019	2018	2017	2016
Republic of Karelia	0.386	0.494	0.810	0.662	0.362	0.465	0.777	0.643	0.932	0.914	0.900	0.906
Republic of Komi	0.441	0.613	0.887	0.632	0.419	0.590	0.886	0.624	1.030	1.034	1.052	1.060
Arkhangelsk region	0.421	0.566	0.815	0.744	0.400	0.538	0.790	0.750	1.015	1.012	1.012	1.007
Vologda region	0.354	0.450	0.681	0.540	0.337	0.420	0.655	0.542	0.809	0.801	0.794	0.800
Kaliningrad region	0.361	0.410	0.626	0.610	0.340	0.392	0.598	0.616	0.802	0.797	0.802	0.815
Leningrad region	0.403	0.371	0.676	0.664	0.391	0.371	0.652	0.660	0.843	0.849	0.854	0.848
Murmansk region	0.549	0.728	0.884	0.830	0.516	0.684	0.858	0.831	1.210	1.198	1.191	1.186
Novgorod region	0.326	0.371	0.651	0.598	0.312	0.369	0.618	0.593	0.770	0.769	0.808	0.813
Pskov region	0.320	0.340	0.628	0.563	0.295	0.315	0.595	0.540	0.733	0.707	0.717	0.706
Saint Petersburg	0.588	0.756	0.729	1.000	0.561	0.715	0.710	1.000	1.254	1.248	1.229	1.214
Nenets AR	1.000	1.000	1.000	0.807	1.000	1.000	1.000	0.847	1.603	1.670	1.640	1.646
Mean value	0.47	0.55	0.76	0.70	0.45	0.53	0.74	0.69	1.00	1.00	1.00	1.00

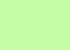
 - Three maximum values of indicators are highlighted.

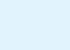
 - The minimum values of indicators are highlighted.

Abbreviation: NWFD - Northwestern Federal District.

### The public finance sector

The standardized values of the indicators reflect the high level of sustainability of the Saint Petersburg, Leningrad, and Kaliningrad regions, which naturally affects the value of the integral sustainability rating of these regions. The regions with a low level of debt sustainability include the Republic of Karelia, the Republic of Komi, and the Pskov region. The rest of the NWFD regions at this stage of the study can be considered as the regions with medium debt sustainability. Due to the developed gradation scale, all regions are well classified into three groups: with high, medium, and low debt sustainability ( Table 6). The integral values of the debt sustainability index for the NWFD regions allow determining the regions with the level of sustainability higher than medium. Over the period of four years, such regions have consistently included Saint Petersburg, the Leningrad, and the Kaliningrad regions.

**Table 6.** The scale of values of the regional debt sustainability index (I _DS_) (the authors’ calculations).

Value range by year				Type of sustainability
2016	2017	2018	2019	
≥1	≥1	≥1	≥1	High
<1; ≥0.32	<1; ≥0.32	<1; ≥0.42	<1; ≥0.44	Medium
< 0.32	< 0.32	< 0.42	< 0.44	Low

### The financial sector

The preliminary calculated standardized indicator values reflect the high level of sustainability of Saint Petersburg, the Murmansk and Kaliningrad regions, and the Nenets Autonomous Region, which will subsequently affect the calculation of the integral sustainability index for the financial sector. For this type of analysis, the regions with a low level of debt sustainability include the Republic of Karelia, the Pskov, Leningrad, and Novgorod regions. The rest of the NWFD regions at this stage of the study can be considered as the regions with medium debt sustainability.

The integral index of financial sustainability shows that the regions with the level of sustainability higher than medium have consistently included Saint Petersburg, the Arkhangelsk, and the Vologda regions for the period of four years. In order to carry out a gradation of the studied regions according to three types of debt sustainability, it is necessary to set up the intervals for such assessment: the regions with the debt sustainability index equal to or greater than one were assigned to the group with high debt sustainability; the subsequent gradation was carried out taking into account the differentiation of annual values within two groups. So, for example, in 2019, the maximum value of the indicator for Saint Petersburg was 1.92, and the minimum value for the Pskov region was 0.58. The average value between 1 and 0.58 is 0.22, therefore, the border of the group with a medium level of the financial sector sustainability is determined from 1 to 0.78, and for groups with low sustainability, the values of the integral indicator will range from 0.78 to 0.

The regions with high financial sustainability include Saint Petersburg, the Arkhangelsk, and Vologda regions. The regions with medium financial sustainability include the Kaliningrad, Leningrad, Murmansk, and Novgorod regions, the Republic of Karelia and the Republic of Komi, and the Nenets Autonomous Region. The only region with low financial sustainability is the Pskov region. Here, it is important to note that the sustainability of the financial sector as a whole is higher than the debt sustainability.

### The sector of corporative and personal finance

The standardized values of indicators for the corporate finance sector reflect the high level of sales profitability of enterprises in the Leningrad and Murmansk regions, and the Republic of Karelia, which will subsequently affect the calculation of the integral index of sustainability of the regional financial system for this sector. The regions with low levels of sales profitability include the Pskov and Kaliningrad regions. The rest of the NWFD regions can be considered as regions with medium sustainability. As for the first indicator of the personal-finance sector, a higher level of per capita money income is accounted for by the Nenets Autonomous Region, the city of Saint Petersburg, the Murmansk, Arkhangelsk regions, and the Republic of Komi. Lower values of the indicator are typical for the Pskov and Novgorod regions. Later on, such differentiation for each standardized indicator will affect the calculation of the integral rating of sector sustainability for the region.

The regions with high sustainability according to the integral index for the corporative finance sector include the Republic of Komi, and the Vologda, Kaliningrad, Novgorod, and Pskov regions. The corporative finance sector of the Nenets Autonomous Region has low sustainability. The corporative finance sectors for the rest of the regions have medium sustainability. In general, the results for the corporative finance sector differ from the financial sector and the sector of debt sustainability, other regions play a leading role in this sector.

It is important to note here that the sustainability of the personal-finance sector is higher than all the sectors of sustainability identified in the study, namely, higher than the sectors of debt, financial, and corporative sustainability. In addition to this, in the personal finance sector, there are no regions with a low level of sustainability, which indicates a high contribution of households to the stable functioning of the region’s financial system. According to the results obtained, the regions with high sustainability include the Republic of Komi, the Arkhangelsk and Murmansk regions, the city of Saint Petersburg, and the Nenets Autonomous Region. The rest of the regions belong to the group of regions with medium sustainability.

At the final stage of assessment, the criteria values of the debt sustainability indicators were determined based on the values of the cluster centroids ( Table 7).

**Table 7.** Results of the study of clusters by regions of the Northwestern Federal District (NWFD) and volume of budget constraints (by the authors’ calculations).

Debt sustainability indicators	Budget constraints (debt limits) for regions with low debt sustainability	
	2018	2019
R1: debt-to-GDP ratio, %	5.04	5.01
R2: debt-to-per capita ratio, rubles per person	45544	30337
R3: debt-to-regional exports ratio, %	104.87	66.28
R4: debt-to-revenue ratio, %	59.39	42.33
R5: debt cost-to-budget expenditures ratio, %	2.83	2.92
R6: debt cost-to-revenue ratio, %	51.5	48.79

The results of the study also identified that the NWFD regions belong to three clusters: cluster 1 - high debt sustainability: Saint Petersburg, the Leningrad region, and the Nenets Autonomous region; cluster 2 - medium debt sustainability: Saint Petersburg, the Vologda, Murmansk, and Kaliningrad regions; and cluster 3 - low debt sustainability: the Republic of Komi, the Republic of Karelia, the Arkhangelsk, Novgorod, and Pskov regions.

## Discussion

The issues of sustainability within the financial system of the Russian Federation are of decisive importance for maintaining the country’s debt security and investment-driven development. The authors suppose that the term ‘stability’ means the invariability of parameters, while the term ‘sustainability’ is used in a broader and more complex sense ( Barykin *et al.,* 2021b). In this article, authors proposed a reliable and understandable methodological apparatus for assessing the sustainability of the financial system of the Russian regions and tested it on data from 11 regions of the NWFD. At the same time, our results need to be validated on a wider data set, for which we can recommend other researchers use our methodology. The chiefs of municipal entities can use the data on the debt sustainability standards to objectively assess the acceptable level of the debt burden and manage the size of the government debt in the event of a decrease in the debt sustainability of budgets. To develop a financial policy aimed at sustainable and stable functioning of the financial system, heads of municipalities can focus on the value of integral indices across the four sectors of sustainability. It is necessary to point out the limitations of the study. The developed methodology has been tested in 11 regions of the NWFD. For a more reliable evidence base, the number of regions should be expanded. The need to increase the sample of Russian regions to confirm the methodology presented in the article is related to the directions of further research. The directions of further research within the framework of the given topic are determined by the need for an annual statistical justification of the differentiated values of the upper limits of the local government debt, taking into account the macroeconomic situation in Russia and the regional priorities of socio-economic development.

The calculations showed the paramount importance of assessing the debt sustainability of the regional financial system since it is debt stability that is the key to stability and sustainability of the entire financial system. According to the results of the study, the debt sustainability of five regions of the NWFD (the Republic of Karelia, the Republic of Komi, and the Vologda, Pskov, and Arkhangelsk regions) is at a low level, as a result of which the authorities need to pursue a balanced debt policy aimed at increasing the sustainability and solvency of the region, reducing the credit risks, focusing on standards of the debt burden obtained during the study of each cluster, as well as the standards established by Art. 107.1 of the Budget Code of the Russian Federation (Official internet portal of legal information (n.d.).

## Conclusions

In general, summing up the results of the assessment of the sustainability of the regional financial system across the four selected sectors, we can note the following:

• When testing the methodology obtained, the authors faced the need for more thorough empirical indicators based on annual statistics, since not all indicators have passed empirical tests for adequacy of the values presented in the collections of values.
• Across the different sectors of sustainability, there is a difference between the regions belonging to one group or another according to the type of sustainability. So, for example, the sustainable regions in the public finance sector (debt sustainability) include the Kaliningrad and Leningrad regions, and the city of Saint Petersburg; in the financial sector, the sustainable regions include the Arkhangelsk and Vologda regions, and the city of Saint Petersburg; in the corporative sector - the Republic of Komi, the Vologda, Kaliningrad, Novgorod, and Pskov regions; in the personal finance sector - the Republic of Komi, the Arkhangelsk, Murmansk regions, the city of Saint Petersburg and the Nenets Autonomous Region. Saint Petersburg is the only region that belongs to the high sustainability group according to each integral index. The personal finance sector is represented by five sustainable regions for the period from 2016 to 2019, which is higher than the number of regions with high sustainability in other sectors and reflects the high contribution of households of the NWFD regions to the sustainability of the financial system.
• In general, the values of the integral indices of financial, corporative, and personal sustainability exceed the value of the integral index of debt sustainability, which indicates higher sustainability of the three sectors in comparison with the public finance sector. Such a conclusion can be made based on the values of integral indicators for different sectors: for example, the tables show that the minimum value of the personal-finance index for the period from 2016 to 2019 for the NWFD regions is not less than 0.5, while the minimum value of the debt sustainability index for the same period is 0.19. It can also be seen that the number of high values of the financial, corporative, and personal integral indices by years for 11 NWFD regions is generally greater than the number of high values of the debt sustainability integral index.
• In the course of the study, the authors also realized the impossibility of calculating a general (aggregate, unified) sustainability index for the four sectors of the financial system, since each sector has its own type of sustainability in terms of homogeneous indicators included in it, which is confirmed by the results of the study. Also, the authors believe that the resulting summary estimate would be difficult to interpret logically. High scores in one of the sectors may overlap with low scores in another sector, as the result of which the region will be assessed as a region with a medium level of sustainability, and such an overall estimate will in no way reflect the detrimental processes of decreasing financial stability in one of the sectors. In our study, this thesis is fully actualized in the assessment of debt sustainability. Therefore, the calculation of the integral index for each sector of sustainability is an advantage of the developed methodology.

## Data availability

Figshare: Sustainability of the regional financial system ENG(1).xlsx. https://doi.org/10.6084/m9.figshare.20278098.v1 ( Barykin *et al.,* 2022).

This project contains the following underlying data:

• Sustainability of the regional financial system ENG (1).xlsx (Data for the calculation of indicators with tabs for ‘Debt of the NWFD regions’, ‘indicators’, ‘index lds’, ‘index lfs’, ‘table’, ‘index lcs’, and ‘index lps’)

Data are available under the terms of the Creative Commons Attribution 4.0 International license (CC-BY 4.0).
