# Supplementary material for: Sustainability of the regional financial system: a case study of the Northwestern Federal District
Source: F1000Res. 2022 Oct 4;11:908. Originally published 2022 Aug 8. [Version 2] doi: 10.12688/f1000research.123197.2 (PMC9714104; doi:10.12688/f1000research.123197.2)
Supplement: Peer review history

# Peer review: Reviewer response for version 2

**DOI:** 10.5256/f1000research.139012.r152333

**Published:** 2022-10-05

**Authors:** Muhammad Ibrar

**Affiliations:** 1 Department of Sociology, University of Swabi, Swabi, Pakistan

Title, abstract, introduction, literature, methods, results, and discussions are informative enough. Last but not least, the paper is well-written, and recommended for indexing.

Is the work clearly and accurately presented and does it cite the current literature?

Yes

If applicable, is the statistical analysis and its interpretation appropriate?

Partly

Are all the source data underlying the results available to ensure full reproducibility?

Partly

Is the study design appropriate and is the work technically sound?

Yes

Are the conclusions drawn adequately supported by the results?

Yes

Are sufficient details of methods and analysis provided to allow replication by others?

Partly

Reviewer Expertise:

Social Sciences i.e. Developmental Research,  Management,/Administration, Public Policy, International Relations, Socio-Cultural Studies, Political Science related Research

I confirm that I have read this submission and believe that I have an appropriate level of expertise to confirm that it is of an acceptable scientific standard.

# Peer review: Reviewer response for version 1

**DOI:** 10.5256/f1000research.135281.r146838

**Published:** 2022-08-23

**Authors:** Alexey Petrovich Zhabko

**Affiliations:** 1 St Petersburg University, St Petersburg, Russian Federation

The paper explores the sustainability of Russian regions’ financial systems in the Northwestern federal district of Russia. I can see that it is an interesting title and deserves some merits. However, there are minor issues to be solved in the manuscript.

**My comments:**

1. The discussion section is too small; it needs to be deepened and expanded, referring to the results of previous studies in assessing the stability of regional financial systems.
2.  How would you explain the choice of indicators in each of the four sectors of the financial system? Why exactly did they go there? The composition of the indicators is well described at the beginning of Section 3, but it is not clear how the choice of indicators within each sector was made. Clarify, please.
3. One of the blocks for assessing the financial system’s stability is debt sustainability. However, the authors do not discuss the concept of a country's debt sustainability in detail. Are our debt management practices similar between developed and developing countries? Especially the issues of debt sustainability of the country's budgets are essential for the federal structure of the state. The article says that the indicators R4, R5, and R6 refer to indicators that regulate the debt burden in the Russian Federation and are regulated by the Budget Code. Still, a more detailed description of holding the debt burden of the budget in the Russian Federation is not presented.
4. What is the fiscal autonomy, in particular, the budgetary autonomy of regional and local governments and the spending autonomy of regions, and what types of transfers, under what conditions and criteria do regional governments (local governments) receive transfers from the federal government (from the regional government) of Russia? It may be worth presenting the debt potential of regions with fiscal transfers of the federal government and comparing it with the debt potential of regions without fiscal transfers of the federal government, as already studied in the international literature in question.
5. The article does not present the foreign trade competencies of Russian regions. Can the governments of Russian regions actively pursue their foreign trade policy, especially exports and imports, without the federal government's approval? What is the share of export revenues of the studied regions in their regional budget? Suppose Russian regions have no such foreign trade competence. In that case, the increase or decrease of a region's exports is not due to its decision but to the federal government’s conclusion. When the federal government decides on exports and gets most of its income from them, then what justification and informative value does indicator 3 (R3) have?

Is the work clearly and accurately presented and does it cite the current literature?

Yes

If applicable, is the statistical analysis and its interpretation appropriate?

Yes

Are all the source data underlying the results available to ensure full reproducibility?

Yes

Is the study design appropriate and is the work technically sound?

Yes

Are the conclusions drawn adequately supported by the results?

Yes

Are sufficient details of methods and analysis provided to allow replication by others?

Yes

Reviewer Expertise:

NA

I confirm that I have read this submission and believe that I have an appropriate level of expertise to confirm that it is of an acceptable scientific standard.

# Response

**Published:** 2022-09-23

**Authors:** Sergey Evgenievich Barykin

**Affiliations:** Graduate School of Service and Trade, Petersburg Polytechnic University, Russia, Russian Federation

1. Thank you. The composition of the indicators is explained by the accumulated experience of other scientists who presented research results and justified the choice of indicators of the sustainability of the financial system, as well as the availability of data collected by the State Statistics Service of the Russian Federation.
2. The distribution of indicators by sector should have been logical. If the indicator characterizes the state of public debt and the microeconomics of the country as a whole, then such indicators were attributed to the debt sustainability sector. If the indicator characterizes the development of the banking system, then it was assigned to the financial sustainability sector. If the indicator describes personal finances (various incomes and expenditures of the population), then it was assigned to the personal sustainability sector. If this indicator characterizes the financial condition of enterprises and corporations in the region, then it was assigned to the corporate sustainability sector.
3. Yes, indeed, the main issue in assessing the sustainability of the financial system is the debt sustainability block. And one of the directions of this topic is the assessment of the acceptable level of public debt. This is an interesting and at the same time quite complex topic. In this paper, we really did not present target values for indicators of the debt level regional budgets, focusing on the methodology for assessing the sustainability of the country's financial system and describing the results obtained, as well as discussing them. At the same time, the authors conduct a study of the safe level of public debt and describe the results in another article. It can be found on the website of the MDPI publishing house (https://www.mdpi.com/2227-7099/10/5/106)
4. The issues of fiscal autonomy are interesting, but in our opinion, it should be the subject of a separate paper. The authors did not conduct research on how the presence or absence of fiscal transfers affects the state of debt sustainability of the financial system of the Russian Federation. Fiscal transfers of the federal government are carried out only under certain conditions and are unevenly distributed across the regions of the Northwestern Federal District. Some regions of the Northwestern Federal District do not have fiscal transfers from the federal budget (federal government) at all. Inclusion in the assessment of the sustainability of regional financial systems is problematic, since there is no publicly available information on the amounts of fiscal transfers.
5. The foreign trade competencies of the Russian regions are quite broad. Since the products for export are produced by enterprises located in a particular region, then most of the income from the sale of export products goes to the regional budget, but not to the federal budget. That is why regions with a high volume of exports have less debt dependence and greater sustainability of regional financial systems. This is confirmed by the results obtained in the paper. Therefore, it is advisable to include R3 indicators in the assessment. At the same time, the value of this indicator R3 in most regions of the Northwestern Federal District (Table 1) is very high, which we assess negatively. This is due to the fact that only a few regions of the Northwestern Federal District sell products for export in high volumes.

# Peer review: Reviewer response for version 1

**DOI:** 10.5256/f1000research.135281.r146837

**Published:** 2022-08-16

**Authors:** Muhammad Ibrar

**Affiliations:** 1 Department of Sociology, University of Swabi, Swabi, Pakistan

Paper title: Sustainability of the regional financial system: a case study of the Northwestern Federal District

 Thank you so much for the opportunity to read and review your paper.  This is a very interesting and informative paper. The authors have put efforts for the best. However, some points need to be cleared in the manuscript.

**Overall comments:**

**1.** In the Methodology for assessing the debt sustainability of the regional budget system section on page 5 of the article, it is said that 45 financial sustainability indicators were selected at the first stage of the study. However, in section 2.3. Materials and Methods on the same page talk about 53 absolute indicators of the socio-economic development of Russian regions, of which 26 relative indicators were selected. How are indicators, absolute and relative indicators related in this study?

**2.** The study says that it is based on data on socio-economic development indicators from 2010 to 2019. What is the reason for the choice of the study period? Why was a more extended period taken, for example, over the last 20-40 years?

**3.** Tables 2,3,4,5 show sustainability indices from 2016 to 2019. Have such indices been calculated for an earlier period, given that macroeconomic indicators have been collected since 2010?

**4.** The study states in Section 2.3, "The study uses the methods of comparison and grouping, correlation analysis, Euclidean distances, calculation of the multivariate mean, and hierarchical cluster analysis." In these methods, for calculating the stability index of the financial system, the use of the multivariate average formula is of crucial importance; therefore, in our opinion, the formula should be given according to the text of the scientific work.

Is the work clearly and accurately presented and does it cite the current literature?

Yes

If applicable, is the statistical analysis and its interpretation appropriate?

Partly

Are all the source data underlying the results available to ensure full reproducibility?

Partly

Is the study design appropriate and is the work technically sound?

Yes

Are the conclusions drawn adequately supported by the results?

Yes

Are sufficient details of methods and analysis provided to allow replication by others?

Partly

Reviewer Expertise:

Social Sciences i.e. Developmental Research,  Management,/Administration, Public Policy, International Relations, Socio-Cultural Studies, Political Science related Research

I confirm that I have read this submission and believe that I have an appropriate level of expertise to confirm that it is of an acceptable scientific standard, however I have significant reservations, as outlined above.

# Response

**Published:** 2022-09-23

**Authors:** Sergey Evgenievich Barykin

**Affiliations:** Graduate School of Service and Trade, Petersburg Polytechnic University, Russia, Russian Federation

**Answer 1:** Thank you for your comment. Indeed, there was a typo in section 2.3, only 45 absolute variables of financial sustainability were selected for research at the initial stage.

 This is the most typical variables of the socio-economic development of Russian regions, collected annually by the Federal Statistics Service.

 Absolute variables and relative indicators are interrelated, since relative indicators were calculated on the basis of absolute variables. For example, when calculating the ratio of the government debt to gross regional product, two absolute variables are used: GRP and public debt.

**Answer 2: **Thank you for your comment. Indeed, the study of debt indicators in the long term is of greater interest. And we also regret it. However, we could not find data on the selected 45 absolute variables in the public domain. This is due to the fact that the set of macroeconomic indicators collected by the Federal State Statistics Service in the Russian Federation changes from time to time, as well as the fact that only in the last decade this information has been presented openly on special resources. We were able to collect the full number of indicators only for the period from 2010 to 2019.

**Answer 3: **Thank you .Yes, the same indicators for four blocks were calculated for the period from 2010 to 2015, but due to the large volume of data, it is not possible to compactly fit the indicators from 2010 to 2019 in one table. Therefore, the results of the study are partially presented.

**Answer 4: **In the paper, in the Data Analysis section, after the quote "The calculation of the integral sustainability indicator was carried out using the multivariate mean formula ...", a link is given to the article where this formula is given on page 77. The entire bibliography of the article is in the list of references and the article is publicly available in the international database Scopus. If, after reading the comment, the reviewer deems it necessary to add the multivariate mean formula to the text of the paper, we will definitely do it.
